# Analysis of Pathogens in Respiratory Tract Infections and Their Effect on Disease Severity: Retrospective Data from a Tertiary Care German Children’s Hospital

**DOI:** 10.3390/children12040438

**Published:** 2025-03-29

**Authors:** Petros Strempas, Heidi Weberruss, Thomas Bollinger, Thomas Rupprecht

**Affiliations:** 1 Medizincampus Oberfranken (MCO), Friedrich-Alexander-Universität Erlangen-Nürnberg (FAU), 91054 Erlangen, Germany; heidi.weberruss@klinikum-bayreuth.de (H.W.); thomas.bollinger@klinikum-bayreuth.de (T.B.); thomas.rupprecht@klinikum-bayreuth.de (T.R.); 2 Children’s Hospital, Klinikum Bayreuth, 95445 Bayreuth, Germany; 3 Department for Microbiology and Laboratory Medicine, Bayreuth General Hospital, 95445 Bayreuth, Germany

**Keywords:** children, respiratory pathogens, viral infections, hospital admission, hospital hygiene

## Abstract

Background: Respiratory tract infections (RTIs) represent a frequent cause of inpatient admission in children’s hospitals, especially in the fall and winter seasons, resulting in major healthcare problems due to a lack of beds. The age and seasonal distribution of each pathogen seem to be multifactorial features that influence the course of infection. Other severity predictors appear to be the length of hospital stay, the presence or absence of oxygen demand, and the value of inflammatory markers. Methods: All inpatients from our children’s hospital between 2021 and 2023 who had a nasopharyngeal swab and presented with RTI symptoms were recruited for this retrospective cohort study. The parameters of interest were age, swab result, month of detection, CRP values, duration of hospitalization, presence of oxygen demand, and comorbidities. The data were analyzed using chi-square tests, paired *t*-tests and regression analysis to determine the associations of differences between the groups. Results: Detection of more than one respiratory pathogen in the same swab, apart from combinations with SARA-CoV-2, influenza, or RS-virus, was not associated with longer hospital stay, higher mean maximal CRP values, or oxygen demand compared to mono-infection with the same pathogens. In contrast, the detection of a pathogen versus no detection could be related to higher rates of oxygen demand and higher CRP values. Conclusions: Since co-infection with more than one virus, excluding those with epidemic potential, was not associated with a more severe course of RTIs, strict patient isolation seems to be dispensable for several viruses, as well as isolation of infected or colonized patients.

## 1. Introduction

### 1.1. Respiratory Infections

Respiratory tract infections (RTIs) are a predominant cause of morbidity, hospitalization, and death in children and can lead to both health and economic consequences for families and society [1,2]. RTIs are infectious diseases that affect the respiratory system and are caused by various pathogens. The clinical spectrum of these medical conditions varies from asymptomatic or mild to severe or fatal. The severity depends on many factors, including the specific pathogen, environmental conditions, and general condition of the patient [3]. Respiratory infections typically develop within hours to days of transmission and include a variety of symptoms, such as fever, cough, sore throat, rhinorrhea, tachypnea, dyspnea, and wheezing [4]. The main pathogens include viruses (rhinoviruses, RSV, coronaviruses, adenoviruses, influenza, and parainfluenza viruses) and bacteria (pneumococci, *Chlamydophila pneumoniae*, and mycoplasmas) [5]. RTIs can be classified into upper respiratory tract infections (URIs) or lower respiratory tract infections (LRIs). The upper respiratory tract consists of the airways from the nostrils to the vocal cords in the larynx, including the paranasal sinuses and the middle ear. The lower respiratory tract includes the continuation of the respiratory tract from the trachea and bronchi to bronchioles and alveoli. RTIs are often not limited to the respiratory tract but can also have systemic effects due to potential spread, microbial toxins, inflammation, or reduced lung function [4,6].

### 1.2. Epidemiological Background

According to the World Health Organization, acute RTIs are responsible for approximately 20% of deaths in children under five years of age [7]. Acute RTIs are among the most common infections in children, particularly during autumn and winter [5]. Some pathogens exhibit an apparent seasonal distribution. According to Price et al. [8], Respiratory Syncytial Virus (RSV) and influenza A virus cases increase from November to January. However, adenoviruses and rhinoviruses can be present year-round, with peak levels in autumn for rhinoviruses and in spring for adenoviruses [9]. In addition, SARS-CoV-2 appears to have a whole-year distribution with several peaks [10], while seasonal coronaviruses are more common in the winter months [11].

Several factors have been identified as potential causes of pathogen seasonality. The main factors include temperature, humidity, environmental changes, and different human behavior patterns depending on the season. As aerosol transmission predominates in indoor areas, a higher RTI incidence is observed during winter [9]. Therefore, between November and March, many clinics nationwide are overloaded. This often leads to a lack of available rooms, especially if strict isolation measures in single rooms are applied for every unique multi-pathogen combination [12].

A retrospective analysis using polymerase chain reaction (PCR) from Senegal showed that viruses are the most common cause of URIs and LRIs in children under 5 years of age [13]. According to the authors, 6% of viral RTIs are associated with bacterial co- or supra-infections, with pneumococci being the predominant bacterial species. Another study by Akkoc et al. highlighted RSV and rhinoviruses as the main causes of LRIs in young children who had to be treated as inpatients at a children’s hospital [14]. In Germany, severe outbreaks of influenza and RS viruses in toddlers in the 2023/2024 season, mainly with influenza and RS- viruses, were documented in kindergartens nationwide [15]. Inflammatory markers, such as CRP, can only be used as a rough severity index, reflecting the inflammatory body process [16,17].

Our retrospective cohort study examined whether there was a connection between the detection of pathogens (or the detection of more than one pathogen) and the severity (length of hospital stay, oxygen administration, level of inflammation parameters) of the hospital stay. From this point of view, we would like to examine the meaningfulness of hygiene tactics, such as strict patient isolation in single rooms, especially during periods when hospitals are overwhelmed by RTI patients. For comparison, a hygiene strategy in which patients with SARS-CoV-2, influenza A/B, and/or RSV were isolated was discussed. With this hygiene concept, children with other viral infections are not isolated but separated from immunosuppressed patients.

Our retrospective cohort study examined whether there was a connection between the detection of pathogens (or the detection of combinations of pathogens) and the severity (length of hospital stay, oxygen administration, level of inflammation parameters) of the hospital stay. From this point of view, we would like to examine which pathogen combinations are associated with more severe outcomes compared with mono-infection. Thus, strict patient isolation, especially during periods of hospital overwhelm, should be discussed according to the combination’s severity. Moreover, this study aims to examine the role of comorbidities in infection’s course and the meaningfulness of the parameters above as outcome indexes.

## 2. Materials and Methods

### 2.1. Study Population

This study is a monocentric retrospective cohort study in which data of pediatric patients from 1 January 2021, to 31 December 2023 were obtained and analyzed. All pediatric patients (aged 0–18 years) who were admitted to the pediatric ward of Klinikum Bayreuth (children’s hospital, Klinikum Bayreuth) and received a nasopharyngeal swab because of RTI symptoms or to rule out the respiratory tract as an infection focus (either a multiplex PCR, see Table 1, or a targeted-quadrable test only for SARS-CoV-2, influenza A/B, and RSV) were included to this study. No admitted pediatric patients were excluded from this study.

We examined the following variables: patient age (in completed years or months), conducted swabs, pathogen detection, detection month, length of stay, oxygen demand (existing or not), CRP values (in mg/L), pathogen incidence by age, and existence of comorbidities. The statistical methods are discussed in Section 2.3.

During 1 January 2021 to 31 December 2023, pathogen detection, length of stay, and data for the detected month were recorded. The data on CRP values, sex, and age distribution related to the inpatients were collected from 1 March 2022 to 31 December 2023. In addition, data on the oxygen supply of inpatient children were obtained from 1 November 2022 to 31 March 2023. An oxygen supply was administered to patients with oxygen saturation < 92%. It should be mentioned that all the patients were strictly isolated in single rooms during this period. The different periods of the study data are attributed to the complicity of data acquisition and are mentioned as a limitation in the discussion. It should be also highlighted that the study population was the same in our cohort study, and variables such as CRP and oxygen demand were recorded and analyzed in a shorter period accordingly.

Regarding the association with comorbidities, we examined inpatients from 1 August 2022 until 31 March 2023 whose admission indication was an RTI with comorbidity known before hospital admission. Children and adolescents with trisomy 21, developmental disorders, formerly premature babies, and children with already diagnosed bronchial asthma or type 1 diabetes mellitus were included in this group. Each of the above-mentioned cases was paired with cases as close as possible in terms of age and pathogen detection, but without comorbidities. Children with more than one germ cell detection were paired with another patient with at least one pathogen in their nasopharyngeal swab.

This study was conducted exclusively in the pediatric wards of “Klinikum Bayreuth” (monocentric). This was a retrospective cohort study in which pathogen detection was compared with other crucial factors from the clinic every day. Patient data were obtained from the hospital hygiene database. All patients or their legal guardians signed a treatment contract upon admission stating that they consented to inpatient treatment. In this study, no innovation or additional therapeutic or diagnostic measures were taken, which would not have been conducted without the study design. All patients were treated as usual, independent of the study, and their inpatient stay data were collected and evaluated after the end of their treatment.

### 2.2. Applied Materials

All multiplex PCR tests were conducted using the “BioFire^®^ Respiratory Panel 2.1 Plus”. (bioMérieux, Marcy l’Etoile, France) to detect viruses and bacteria [18,19]. Molecular diagnostics of targeted (quadruble) PCR (“Xpert^®^ Xpress CoV-2/Flu/RSV plus”, Cepheid, Krefeld, Germany) also took place in our laboratory and were based on RNA detection of the four most common respiratory pathogens of severe RTI (influenza A/B, SARS-CoV-2, RSV).

All swabs (“UTM^®^ Universal Transport Medium™”, Copan, Brescia, Italy) were received during the patients’ hospital stay, and no extra swabs or other diagnostic or therapeutic measures were taken exclusively for the study objectives. No complications related to the nasopharyngeal swabs were reported.

### 2.3. Statistics

The data were assessed using Microsoft Excel (Microsoft Office 16; Redmond, WA, USA). Statistical analysis was performed using SPSS for Windows Version 28.0.0.0 (Chicago, IL, USA).

After testing for normal distribution using the Kolmogorov–Smirnov test, we examined the differences in hospital stay in days and max. CRP values between different pathogen combinations via regression analysis (CATREG, regression go categorical data). Chi-square tests were performed to compare the difference in the existence of oxygen demand between patients with or without pathogen detection and between patients with detection of one or more pathogens. Furthermore, we used the chi-square test to correlate the difference between the expected and observed monthly detection rates of all pathogens. Cramer’s V was estimated as an index for interpretation of the association between the examined parameters when the chi-square test was used. According to their values, Cramer’s V can be used to evaluate the strength of the association [20,21]. The confidence interval was 95% for each case.

To control for the impact of comorbidities, a paired *t*-test was used to analyze the difference in metric parameters between the paired groups (CRP values, length of stay), as both groups with comorbidities and the control group consisted of more than 30 cases and a normal distribution could be assumed [22]. McNemar’s test was used to control for differences in non-metric parameters (oxygen demand) between the groups.

The maximum CRP (in mg/L) of all inpatients from 1 March 2022 to 31 December 2023 was documented for the purposes of this study. To investigate this relationship, the mean maximum CRP level of the patients was calculated. If the laboratory value of CRP was <0.6 mg/L, then it was designated as “0” for efficient calculation of the mean. If there were different decimal places due to different measured values (mg/dL and mg/L) from our laboratory, all decimal places were omitted from the calculation of the mean. It is worth mentioning that all CRP values were converted to mg/L for a universal calculation.

A sensitivity analysis was performed considering this study’s limitations [23]. Our hospital database contains all conducted swabs during the study period, so missing data can be minimized as much as possible. All metric parameters were clearly defined before the study began. Minor deviations in protocol or outcome definitions could lead to different results as our study consisted of definite parameters: population and swab results. All data were retained and analyzed after patient discharge so no outcome biases could occur. Controls for normal distribution were performed, and the correlations were statistically analyzed with other possible tests, which provided similar results (Mann–Whitney U, *t*-test for independent samples). Further analyses of age, sex, or other subgroups were not performed, and this is a limitation of this study.

## 3. Results

### 3.1. Epidemiological Surveillance

#### 3.1.1. Study Population

During the period of 2021–2023, 4884 nasopharyngeal results for 4100 patient cases were collected (2698 multiplex and 2186 targeted PCR). Of these, 1929 results from multiplex PCR and 391 from targeted PCR revealed pathogen detection (positive swabs). The exact PCR indication (presence of one specific or multiple symptoms ruling out or verifying a suspicion of an RTI) was not controlled for among the study population. The mean length of hospital stay in the study population was 6.54 hospital days. Age data were collected only in the period from 1 March 2022 to 31 December 2023; therefore, the mean age of the study population could not be reported. Other anthropometric data were not evaluated in this study.

#### 3.1.2. Incidence of the Most Common Pathogens in Childhood

**Figure 1:**
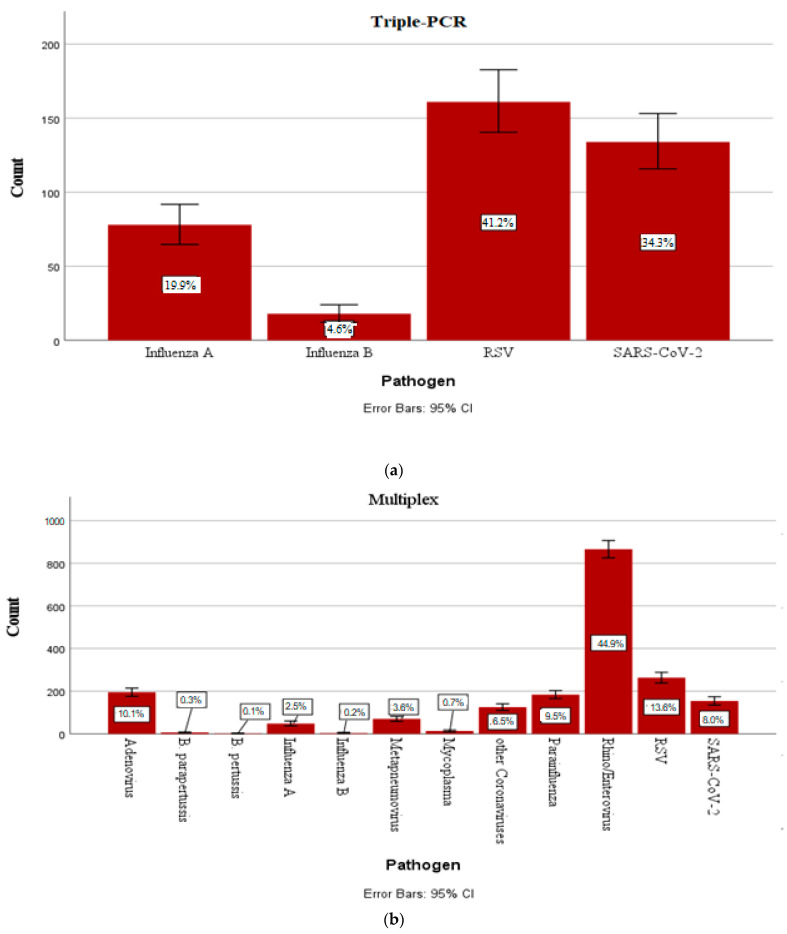
(**a**) Percent detection rate of each pathogen detectable using quadruple PCR among positive swabs in the period of January 2021 to December 2023 with error bars showing 95% confidence intervals. (**b**) Percent detection rate of each pathogen detectable using multiplex PCR among positive swabs in the period of January 2021 to December 2023 with error bars showing 95% confidence interval.

Of the pathogens detected using targeted PCR, RSV and SARS-CoV-2 showed the highest incidences (41.2% and 34.3% of the positive smears). Figure 1a summarizes the number of cases of each pathogen as well as the percentage detection rate from 01/2021 to 12/2023 with error bars showing the 95% confidence interval.

Regarding multiplex PCR, rhino/enteroviruses appeared to be the pathogens with the highest incidence (44.9% of positive swabs with one or more pathogens). RSV and adenoviruses were the second and third most-frequently detected viruses (13.6% and 10.1% of the positive cases, respectively). The incidence and percentage detection rate of all detectable bacteria/viruses with error bars showing 95% confidence intervals are given in Figure 1b. The term “other coronaviruses” consists of seasonal coronaviruses (coronavirus NL63/229E/OC43/HKU1) and “parainfluenza viruses” of parainfluenza viruses 1–4, which can be identified using multiplex PCR.

In the case of detection of more than one pathogen, rhino/enteroviruses (74% of co-infection cases) were involved in most combinations, followed by adenovirus (30%) and parainfluenza viruses (24%). Furthermore, the combinations of rhino/enterovirus and RSV were the most often observed among all possible combinations (22% of co-infection cases), while the combination of adeno and rhino/enterovirus was in second place (15.2% of co-infection cases).

#### 3.1.3. Pathogen Incidence and Seasonality

**Figure 2:**
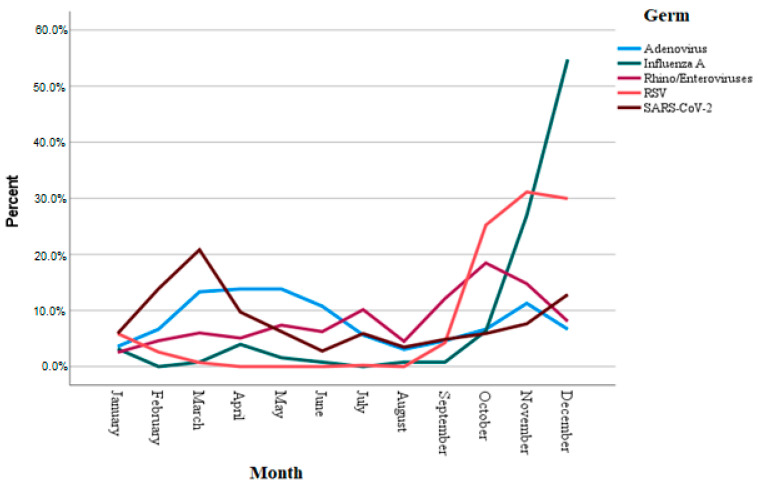
Monthly distribution of the five pathogens with the highest detection rate.

Regarding the seasonal distribution of each pathogen, the monthly detection percentage of the five pathogens with the highest incidence (adenovirus, rhino/enteroviruses, SARS-CoV, and influenza A virus) is shown in Figure 2.

#### 3.1.4. Pathogen Incidence by Age

**Figure 3:**
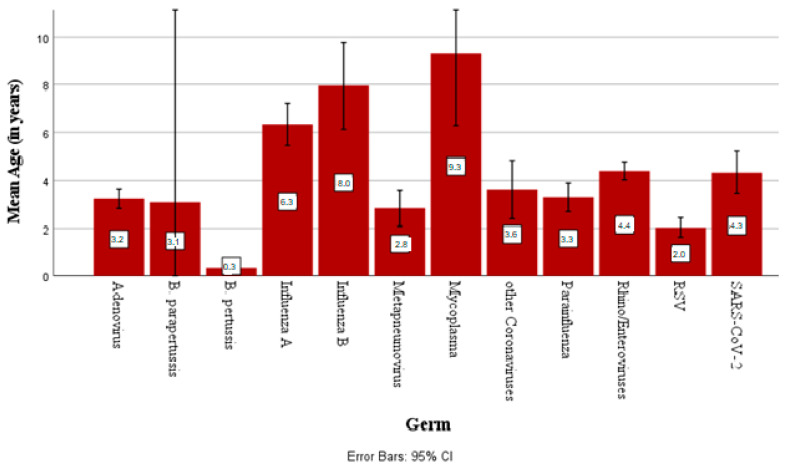
Mean age of detection of each respiratory germ with 95% confidence interval.

Among the detectable pathogens, mycoplasma was the germ with the highest mean patient age (9.3 years ± 3.9), while the lowest was observed for RSV (2 years ± 2.9). Figure 3 shows the mean age at detection for every respiratory pathogen.

Regarding further grouping into age groups (0–6, 6–12, >12 years), most inpatient cases affected children up to 6 years of age, especially during their first year of life. In the age group of 0–6-year-olds, rhino/enteroviruses (34.6% of positive cases) were the pathogens with the highest incidence, followed by RSV (18.8%). RSV was the virus accounting for most cases in infants <1 year of age (30.3% of positive cases), but rhino/enteroviruses (42%) are the predominant pathogens in the neonatal period (children up to 28 days of life).

### 3.2. Statistical Associations Between Parameters

#### 3.2.1. Association Between Length of Stay and Pathogen Detection

SARS-CoV-2 appeared to be the pathogen with the longest length of stay (5.60 ± 9.04 inpatient days), followed by RSV and B. pertussis, while the shortest stay was observed for influenza virus B (3.71 ± 1.88 days).

Table 2 shows the mean length of hospital stay for all detectable pathogens.

Regarding simultaneous detection of more than one pathogen, Table 3 summarizes the incidence rate and the mean length of stay of each pathogen combination with the 95% confidence interval.

From 2021 to 2023, the relationship between the detection of one or more pathogens and the length of stay of inpatients was controlled. More specifically, regression for categorical data (CATREG) showed that the combinations of rhino/entero- with adenoviruses, rhino/enterovirus with RSV and a triple-detection of parainfluenza virus, and rhino/enterovirus and RSV were not associated with statically significant longer inpatient stays than the detection of each virus alone (*p* > 0.05).

Contrarily, co-infection with influenza A virus and RSV resulted in significantly longer hospitalization than the detection of influenza A (*p* 0.01), but not longer than RSV.

#### 3.2.2. Association of Oxygen Demand with Pathogen Detection

Among the 808 collected patient cases, oxygen requirements were analyzed (194 with and 614 without O_2_ supply). It appeared that patients with pathogen detection in their respiratory samples developed the need for oxygen supply more frequently than those without (*p* < 0.001, Cramer’s V 0.227). Regarding the cases with detection of more than one pathogen in the same swab, Table 4 shows the oxygen demand in each pathogen combination detected.

In addition, we controlled the difference in oxygen demand between patients with co-infection and mono-infection with one of the viruses involved. The chi-square test showed that the combination of influenza A and RSV was more often associated with oxygen demand than the detection of influenza A (*p* < 0.01), but not when compared to the single detection of RSV (*p* 0.44). Moreover, the simultaneous detection of RS and rhino/enteroviruses required oxygen administration statistically significant more often compared to mono-infection with a rhino/enterovirus (*p* 0.03), but not more often than RSV infection.

On the other hand, co-infection with adeno-/rhinoviruses, seasonal corona-/rhinoviruses, parainfluenza-/rhinoviruses, or parainfluenza-/adenoviruses had no significant difference (*p* > 0.05) in oxygen demand compared with the detection of only one of these viruses.

#### 3.2.3. Association of Oxygen Demand with Duration of Inpatient Stay

After the regression analysis for categorical data between the two groups, it was shown that patients with O_2_ requirement remained longer in the hospital compared to patients without oxygen supply (*p* < 0.001, power 100%).

#### 3.2.4. Association of CRP Values with Pathogen Detection

A regression analysis of the mean of the maximum measured CRP (in mg/L) during the hospital stay showed that children with positive smears (detection of at least one pathogen) did not have statistically significantly (*p* 0.09) higher CRP levels compared to children with a negative PCR result.

For a report on the detection of multiple pathogens, Table 5 summarizes the mean CRP value of each pathogen combination detected via nasopharyngeal swab.

According to the regression analysis for the categoric data, the simultaneous detection of parainfluenza viruses with RSV brought a higher mean of CRP values (*p* 0.026 < 0.05) in comparison with a mono-infection with a parainfluenza virus. Apart from this combination, there was no statistically significant difference in mean CRP values between every co-infection and a monoinfection with each of the combination’s viruses/bacteria (*p* > 0.05).

#### 3.2.5. Associations with Comorbidities

The paired *t*-test and McNemar’s test revealed that in the group with comorbidities, the mean length of stay was significantly longer (*p* < 0.05), and the presence of oxygen demand was more frequent (*p* 0.049 < 0.05). In contrast, there was no statistically significant difference in the maximum CRP values (*p* = 0.68) between the group with existing comorbidities and the group with non-existing comorbidities.

## 4. Discussion

According to our epidemiological surveillance, RSV and rhino/enteroviruses (a heterogeneous group of various viruses) [24] appear to be the most common causes of inpatient admission in children with respiratory infections. Furthermore, SARS-CoV-2 has been associated with a longer hospital stay than other viruses, whereas RSV predominately affects children in their first year of life. These findings seem compatible with other surveys worldwide, showing that both RSV and SARS-CoV-2 often lead to outbreaks, mainly in the fall and winter months [15], which is a critical health problem in childhood and infancy, overwhelming pediatric hospitals [25]. Children in their first year of life had the most inpatient admissions, which is congruent with other studies and could be attributed to the immaturity of the immune system in younger children [26]. In addition, newborns seem to be more vulnerable to rhino/enteroviruses than other respiratory pathogens, a period of life that requires close observation from healthcare providers, as the risk of early- or late-onset neonatal sepsis has an eventual lethal impact on the child’s health [27].

With regard to seasonal distribution, most respiratory pathogens, especially those with epidemic outbreak dynamics (SARS-CoV-2, RSV, rhino/enteroviruses, influenza), displayed a peak incidence in either the autumn or winter months (often characterized as the “cold season”), whereas adenoviruses presented a higher-than-expected detection rate in spring (March to May). The seasonality of each respiratory virus is suggested to be a multifactorial feature [9].

Beyond the epidemiological findings, an association control between the results of the nasopharyngeal swab and several parameters, which can be used as outcome indices, was conducted during this study. It was shown that co-infection with RS- or influenza A virus could lead to longer inpatient stay or more frequent requirements for oxygen supply than a mono-infection with each pathogen involved.

Contrarily, apart from the viruses with epidemic potential (SARS-CoV, influenza, RSV) combinations of all other possible pathogens in a patient were not associated with longer hospitalization, more frequent oxygen demand (features that seem to be important for RTIs), or increased values of inflammatory markers of the affected children compared to children or adolescents with a detection of only one of the combination’s pathogens. Hence, we did not find any unfavorable severity features among patients with simultaneous colonization/infection with these pathogens at the same time.

The number of different combinations of pathogens in multiplex PCR can be calculated based on the binomial coefficient n over k, where n is the number of pathogens detectable in the test and k is the number of truly positive findings in one patient. According to these calculations, the simultaneous detection of two different pathogens from our multiplex PCR can lead to 190 different combination, and the detection of three different pathogens can lead to 1140, meaning the rate of single-bedroom isolation of patients with more than one pathogen will be close to 100%. It is obvious that the availability of a single bed for every patient in seasons with a high incidence of respiratory infections is barely impossible.

In contrast, a longer hospital stay was observed in children with negative PCR tests. A possible explanation seems to be bacterial upper or lower respiratory infections (e.g., streptococcal pharyngitis, pneumococcal, or legionella pneumonia), which cannot be detected with our PCR tests and are often associated with a more severe course. Confounding conditions such as the existence of another admission indication (e.g., infection of unknown origin to rule out a respiratory infection, patients with underlying diseases who receive screening PCR) and the conducting of PCR in inpatients with a long hospital stay like premature babies as a part of hygiene-based screening in order to prevent nosocomial outbreaks could also be a reason for the longer hospital stay of inpatients with negative PCR results. Finally, the uncertainty of healthcare professionals who treat children with negative PCR tests could lead to a longer hospital stay for further diagnostics, since these patients often have no established diagnosis.

Moreover, it should be highlighted that the presence of comorbidities, especially chronic lung diseases such as bronchopulmonary dysplasia (BPD) or bronchial asthma, appears to be a risk factor associated with more severe outcomes of RTIs [28]. Since the existence of oxygen demand has been significantly associated with longer hospital stays, oxygen supply could be used as a severity predictor of inpatient stay, because a longer average stay duration is observed in such patients. Thus, isolation measures in this group could be meaningful, as co-infection may be associated with more severe clinical consequences.

The different control periods of each parameter and the application of two PCR tests with different detection spectra could be referred to as a limitation of this study. The group of children with comorbidities consisted of cases with heterogeneous chronic disorders, and there were no patients with oncological or cardiac diseases, as our children’s hospital does not treat these patients on a regular basis. Furthermore, potential biases in testing frequency (as the viruses detected via quadruple PCR can be more frequently observed) or in the severity course and the treatment of different pathogen groups (since the swab result is known to the healthcare professionals) could also have occurred.

All information was collected after the discharge of the patients so that their treatment strategies could be completely independent of our retrospective analysis.

Preventive measures can play a very important role in the control of RTIs when they are applied on time [29]. Well-known and studied measures, such as proper handwashing or wearing of a face mask, have been reported to provide a more advantageous effect in reducing or avoiding cross-infection in hospitals [29,30]. It is obvious that more studies on RTIs in childhood need to be conducted to gather knowledge and experience.

## 5. Conclusions

As every clinical outcome can be based on the effective organization and administration of the health care unit, it seems to be important to acquire accurate epidemiological and severity indexes to efficiently manage every case of hospital overwhelm [31].

As the detection of more than one virus (or bacteria), excluding RSV, influenza virus, and SARS-CoV-2, has not been shown to be associated with a worsened outcome in our pediatric wards, it can be suggested that strict patient isolation is not necessary for every single pathogen. This is an important result of this study, especially in periods of seasonal RTI when pediatric hospitals are overwhelmed and single rooms are scarce [32,33]. However, strict patient isolation could be useful in children with potentially more severe outcomes due to existing comorbidities and in viruses with a high epidemic potential (RSV, influenza virus, and SARS-CoV-2).

## Figures and Tables

**Table 1:** Detectable pathogens using multiplex PCR.

Viruses	Influenza virus A/B, RSV, metapneumovirus, parainfluenza virus 1–4, adenovirus, coronavirus NL63/229E/OC43/HKU1, rhino/enterovirus, MERS coronavirus, SARS-CoV-2
Bacteria	*Mycoplasma pneumoniae*, *Chlamydophila pneumoniae*, *Bordetella pertussis*, *Bordetella parapertussis*

**Table 2:** Length of stay according to detected respiratory pathogen.

Pathogen	Count	Mean Length of Stay (Days)	Standard Deviation (95% CI)
Adenovirus	195	4.37	2.20
Other coronaviruses	125	4.22	2.69
*B. parapertussis*	6	5.17	1.72
*B. pertussis*	2	5.5	3.54
Influenza A	126	3.96	2.46
Influenza B	21	3.71	1.88
Negative	2564	8.08	11.88
Metapneumovirus	70	4.70	2.54
Mycoplasma	13	4.15	1.40
Parainfluenza	184	4.19	3.11
Rhino/enteroviruses	866	5.03	7.64
RSV	424	5.5	2.74
SARS-CoV-2	288	5.60	9.04
Total	4884	6.54	9.28

**Table 3:** Frequency, incidence rate, and mean length of stay in inpatient days of each pathogen combination detected with 95% confidence interval.

Pathogen Combinations	Count	Incidence Rate (%)	Mean Length of Stay in Days (95% CI)
Adenovirus/*B. parapertussis*	1	0.3	8.00
Adenovirus/other coronaviruses	11	3.4	3.90 ± 1.86
Adenovirus/other coronaviruses/parainfluenzavirus	1	1.3	4.00
Adenovirus/other coronaviruses/parainfluenzavirus/rhino/enterovirus/RSV	1	0.3	7.00
Adenovirus/other coronaviruses/parainfluenzavirus/rhino/enterovirus/RSV	1	0.3	3.00
Adenovirus/metapneumovirus	1	0.3	7.00
Adenovirus/metapneumovirus/rhino/enterovirus	1	0.3	3.00
Adenovirus/parainfluenzavirus	11	3.4	4.54 ± 1.57
Adenovirus/parainfluenzavirus/rhino/enterovirus	4	1.2	2.00
Adenovirus/rhino/enterovirus	49	15.2	4.53 ± 2.12
Adenovirus/rhino/enterovirus/RSV	6	1.9	4.83 ± 2.48
Adenovirus/rhino/enterovirus/SARS-CoV2	1	0.3	4.00
Adenovirus/RSV	3	0.9	6.33 ± 2.08
Adenovirus/SARS-CoV2	4	1.2	3.50 ± 1.73
*B. parapertussis*/rhino/enterovirus	2	0.6	5.50 ± 0.70
*B. parapertussis*/RSV	1	0.3	4.00
*B. pertussis*/rhino/enterovirus	1	0.3	3.00
Other coronaviruses/influenza A	1	0.3	2.00
Other coronaviruses/other coronaviruses	1	0.3	3.00
Other coronaviruses/metapneumovirus	1	0.3	13.00
Other coronaviruses/parainfluenzavirus	2	0.6	3.00
Other coronaviruses/parainfluenzavirus/rhino/enterovirus	2	0.6	4.50
Other coronaviruses/parainfluenzavirus/rhino/enterovirus/SARS-CoV2	1	0.3	3.00
Other coronaviruses/rhino/enterovirus	20	6.2	3.95 ± 2.06
Other coronaviruses/rhino/enterovirus/RSV	3	0.9	3.00 ± 1.00
Other coronaviruses/RSV	9	2.8	4.22 ± 2.53
Other coronaviruses/SARS-CoV2	1	0.3	4.00
Other coronaviruses/RSV	1	0.3	3.00
Influenza A/parainfluenzavirus	1	0.3	2.00
Influenza A/RSV	9	2.8	6.33 ± 1.93
Influenza A/SARS-CoV2	5	1.5	3.60 ± 0.89
Influenza A/rhino/enterovirus	5	1.5	3.80 ± 1.79
Influenza B/SARS-CoV2	1	0.3	4.00
Metapneumovirus/rhino/enterovirus	7	2.2	5.86 ± 4.67
Metapneumovirus/RSV	1	0.3	6.00
Metapneumovirus/SARS-CoV2	6	1.9	4.33 ± 1.50
Mycoplasma/rhino/enterovirus	4	1.2	3.75 ± 0.96
Mycoplasma/SARS-CoV2	1	0.3	4.00
Parainfluenzavirus/parainfluenzavirus	1	0.3	6.00
Parainfluenzavirus/rhino/enterovirus	37	11.5	3.95 ± 2.24
Parainfluenzavirus/rhino/enterovirus/RSV	4	1.2	6.50 ± 1.91
Parainfluenzavirus/rhino/enterovirus/SARS-CoV2	1	0.3	9.00
Parainfluenzavirus/RSV	4	1.2	4.50 ± 1.00
Parainfluenzavirus/SARS-CoV2	2	0.6	3.50 ± 3.54
Rhino/enterovirus/RSV	71	22.0	5.94 ± 3.52
Rhino/enterovirus/SARS-CoV2	16	5.0	3.18 ± 0.98
RSV/SARS-CoV2	6	1.9	3.83 ± 1.83
Total	323	100.0	4.67 ± 2.62

**Table 4:** Detected pathogen combinations with observed oxygen demand.

	Oxygen Demand		
Pathogen Combinations	Yes	No	Total
Adenovirus/other coronaviruses	0	1	1
Adenovirus/other coronaviruses/parainfluenzavirus	0	1	1
Adenovirus/metapneumovirus	1	0	1
Adenovirus/parainfluenzavirus	0	3	3
Adenovirus/parainfluenzavirus/rhino/enterovirus	0	1	1
Adenovirus/rhino/enterovirus	1	9	10
Adenovirus/rhino/enterovirus/RSV	1	1	2
*B. parapertussis*/rhino/enterovirus	1	1	2
*B. parapertussis*/RSV	0	1	1
*B. pertussis*/rhino/enterovirus	0	1	1
Other coronaviruses/ influenza A	0	1	1
Other coronaviruses/other coronaviruses	0	1	1
Other coronaviruses/metapneumovirus	1	0	1
Other coronaviruses/rhino/enterovirus	0	2	2
Other coronaviruses/RSV	0	1	1
Influenza A/RSV	7	2	9
Influenza A/SARS-CoV2	0	4	4
Influenza A/rhino/enterovirus	0	5	5
Influenza B/SARS-CoV2	1	0	1
Metapneumovirus/rhino/enterovirus	1	3	4
Metapneumovirus/RSV	1	0	1
Metapneumovirus/SARS-CoV2	1	0	1
Parainfluenzavirus/parainfluenzavirus	1	0	1
Parainfluenzavirus/rhino/enterovirus	1	15	16
Parainfluenzavirus/rhino/enterovirus/RSV	1	0	1
Parainfluenzavirus/RSV	2	0	2
Rhino/enterovirus/RSV	9	6	15
Rhino/enterovirus/SARS-CoV2	0	2	2
RSV/SARS-CoV2	1	2	3
Total	31	63	94

**Table 5:** Mean values of max. CRP of each pathogen detection with 95% CI.

Pathogen Combinations	Mean Max. CRP (in mg/L) 95% CI
Adenovirus/other coronaviruses	18.50 ± 19.41
Adenovirus/other coronaviruses/parainfluenzavirus	49.0
Adenovirus/metapneumovirus	4.0
Adenovirus/parainfluenzavirus	50.42 ± 51.85
Adenovirus/parainfluenzavirus/rhino/enterovirus	11.50 ± 13.51
Adenovirus/rhino/enterovirus	41.17 ± 62.17
Adenovirus/rhino/enterovirus/RSV	62.00 ± 57.02
Adenovirus/rhino/enterovirus/SARS-CoV2	10.20
Adenovirus/RSV	33.45 ± 4.17
Adenovirus/SARS-CoV2	25.25 ± 29.36
*B. parapertussis*/rhino/enterovirus	141.00 ± 192.33
*B. parapertussis*/RSV	1.00
Other coronaviruses/influenza A	1.00
Other coronaviruses/other coronaviruses	1.00
Other coronaviruses/parainfluenzavirus	5.00
Other coronaviruses/rhino/enterovirus	32.29 ± 50.80
Other coronaviruses/RSV	9.00
Influenza A/parainfluenzavirus	36.00
Influenza A/RSV	40.9 ± 60.95
Influenza A/SARS-CoV2	8.60 ± 7.37
Influenza A/rhino/enterovirus	13.80 ± 14.92
Influenza B/SARS-CoV2	49.00
Metapneumovirus/rhino/enterovirus	12.80 ± 13.81
Metapneumovirus/RSV	32.00
Metapneumovirus/SARS-CoV2	7.83 ± 5.27
Mycoplasma/rhino/enterovirus	59.75 ± 35.07
Mycoplasma/SARS-CoV2	16.10
Parainfluenzavirus/parainfluenzavirus	33.00
Parainfluenzavirus/rhino/enterovirus	16.44 ± 21.75
Parainfluenzavirus/rhino/enterovirusRSV	12.60 ± 14.99
Parainfluenzavirus/rhino/enterovirus/SARS-CoV2	12.00
Parainfluenzavirus/RSV	56.00 ± 29.70
Parainnfluenzavirus/SARS-CoV2	2.50 ± 0.71
Rhino/enterovirus/RSV	39.21 ± 51.04
Rhino/enterovirus/SARS-CoV2	10.04 ± 10.29
RSV/SARS-CoV2	12.10 ± 21.31
Total	29.38 ± 45.80

## Data Availability

The original data presented in the study are openly available in https://figshare.com/articles/dataset/Supporting_data/28713062, accessed on 27 March 2025.

## Abbreviations

The following abbreviations are used in this manuscript:

CRP	C-reactive protein
CATREG	Regression go categorical data
CI	Confidence interval
e.g.	Exempli gratia, for example
LTIs	Lower respiratory tract infections
MERS	Middle East Respiratory Syndrome
mg/L	Milligrams per liter
PCR	Polymerase chain reaction
RSV	Respiratory syncytial virus
RTIs	Respiratory tract infections
SARS-CoV-2	Severe acute respiratory syndrome coronavirus 2
UTIs	Upper respiratory tract infections
